# Kiwifruit Allergy in Children: Characterization of Main Allergens and Patterns of Recognition

**DOI:** 10.3390/children2040424

**Published:** 2015-10-19

**Authors:** Ana Moreno Álvarez, Leticia Vila Sexto, Luda Bardina, Galina Grishina, Hugh. A. Sampson

**Affiliations:** 1 Department of Pediatrics, Children’s Hospital Teresa Herrera, A Coruña University Hospital, Xubias de Arriba 84, 15006 La Coruña, Spain; E-Mail: leticia.vila.sexto@sergas.es (L.S.); 2 Division of Pediatric Allergy and Immunology and the Jaffe Food Allergy Institute, The Mount Sinai School of Medicine, New York, 10029-6574, NY, USA; E-Mails: luda.bardina@mssm.edu (L.B.); galina.grishina@mssm.edu (G.G.); hugh.sampson@mssm.edu (H.A.S.)

**Keywords:** actinidin, SDS-PAGE immunoblotting, pediatric, kiwifruit, food allergy

## Abstract

Kiwifruit allergy has been described mostly in the adult population, but immunoglobulin (Ig)E-mediated allergic reactions to kiwifruit appear to be occurring more frequently in children. To date, 13 allergens from kiwifruit have been identified. Our aim was to identify kiwifruit allergens in a kiwifruit allergic-pediatric population, describing clinical manifestations and patterns of recognition. Twenty-four children were included. Diagnosis of kiwifruit allergy was based on compatible clinical manifestations and demonstration of specific IgE by skin prick test (SPT) and/or serum-specific IgE determination. SDS-PAGE and immunoblotting were performed with kiwifruit extract, and proteins of interest were further analyzed by mass spectrometry/mass spectrometry. For component-resolved *in vitro* diagnosis, sera of kiwifruit-allergic patients were analyzed by an allergen microarray assay. Act d 1 and Act d 2 were bound by IgE from 15 of 24 children. Two children with systemic manifestations recognized a protein of 15 kDa, homologous to Act d 5. Act d 1 was the allergen with the highest frequency of recognition on microarray chip, followed by Act d 2 and Act d 8. Kiwifruit allergic children develop systemic reactions most frequently following ingestion compared to adults. Act d 1 and Act d 2 are major allergens in the pediatric age group.

## 1. Introduction

Kiwifruit (*Actinidia*) is a plant native to the Yangtze Valley that at the beginning of the nineteenth century grew wild in China [1]. Seeds of the green-fleshed kiwifruit, *Actinidia deliciosa*, were introduced into New Zealand in 1904 and exports to Europe and the USA started in 1962.

Allergic reactions to kiwifruit were first described in 1981 [2]. Since then, there have been an increasing number of publications on kiwifruit allergy. In Spain, the prevalence of kiwifruit sensitization has been estimated at 1.8% of the general population [3].

From the few studies describing kiwifruit allergy in pediatric populations, it appears that children are more likely to react on the first known exposure and more frequently develop systemic manifestations than adults [4]. Additionally, although kiwifruit allergy is most frequently associated with grass and birch pollen allergies [5], children are frequently mono-sensitized to kiwifruit, suggesting a role of primary digestive tract sensitization and a different pattern of IgE recognition of kiwifruit proteins than in adults.

To date, thirteen allergens have been identified in kiwifruit. Act d 1, called actinidin, represents about 50% of the total soluble protein content [6] and is considered a major allergen. The relevance of Act d 2, a thaumatin-like protein and Act d 3, a 45 kDa glycoallergen, [7,8,9] has yet to be elucidated. Act d 4 acts as an inhibitor of cysteine proteinases [10], with unclear clinical relevance. Act d 5, Kiwellin, is a cystein-rich protein that may undergo *in vivo* and *in vitro* proteolytic processing by kiwifruit actinidin, thus splitting in two additional proteins, KiTH and kissper [11]. Act d 6, a pectin methylesterase inhibitor that may be involved in the regulation of the fruit ripening, and Act d 7, a pectin methylesterase, seem to be recognized by a minority of allergic patients [12]; Act d 8, corresponds to a pathogenesis-related protein class 10 (PR-10) homologous to the major allergen of birch pollen Bet v 1 [13]. Other minor allergens from kiwifruit are Act d 9, a 14 kDa profilin, Act d 10, a nonspecific lipid transfer protein (LTP) [10] and Act d 11 a major latex protein/ripening-related protein that cross reacts with members from the PR-10 family [14]. Recently, two novel allergens contained in kiwifruit seeds have been described and characterized: Act d 12, a 51 kDa 11S globulin that represents a major allergen and Act d 13, a 12 kDa 2S albumin, which is a minor allergen. Both proteins share common epitopes from peanut and tree nuts, suggesting that both allergens might be involved in cross-reactivity with those allergenic sources [15].

Allergen component-resolved diagnostics (CRD) is an emerging *in vitro* tool for the diagnosis of food allergies. This method utilizes purified or recombinant allergens for identification of specific molecules causing sensitization or clinical allergy. CRD is becoming more interesting in this field, since it offers information regarding the probability of local oral *vs.* systemic allergic reactions following food ingestion, based on the specific molecule recognition patterns [16].

Our aim was to describe clinical and epidemiological characteristics of children allergic to kiwifruit as well as to identify major allergens from kiwifruit in this age group.

## 2. Methods

### 2.1. Patients

Children with kiwifruit allergy were recruited from the Pediatric Allergy Unit, at the University Hospital of La Coruña (Spain). Diagnosis of kiwifruit allergy was based on a convincing history of an objective allergic reaction after green kiwifruit ingestion in at least two occasions and demonstration of specific IgE to kiwifruit by skin prick test (SPT) and/or serum specific IgE. In patients with questionable symptoms (mild or subjective reactions) or when several foods were implicated, a single-blind food-challenge with kiwifruit was performed.

### 2.2. Ethical Considerations

The study was approved by the local Ethics Committee (approval number: 2010/423). All subjects provided written informed consent before enrollment into the study.

### 2.3. Skin Tests

SPT were performed with commercial extract (Leti Laboratories, Madrid, Spain) and with fresh kiwifruit pulp. The reaction was regarded as positive if the mean wheal diameter was at least 3 mm greater than the negative control (saline solution 0.9%).

### 2.4. Specific IgE Determination

Specific IgE to green kiwifruit was determined by the CAP system FEIA (Thermofisher Scientific, Barcelona, Spain), and was considered positive when greater than 0.35 kUA/L.

### 2.5. Preparation of Kiwifruit Protein Extract

Green kiwifruit (*Actinidia deliciosa* cv. Hayward) was peeled, cut into pieces, and frozen at −80 °C within 30 min. Frozen kiwifruit pulp was homogenized and mixed with phosphate buffered saline (PBS) containing a protease inhibitor cocktail (Roche, Penzberg, Germany) (1:1 (w:v)). After centrifugation for 30 min at 20.000 g and 4 °C, the supernatant was dialyzed against PBS over 12h at 4 °C. The extract was then aliquoted and stored at −80 °C. The protein concentration was determined by the Coomassie Plus Protein Assay (Pierce, Rockford, IL, USA) according to the manufacturer’s protocol.

### 2.6. Sodium Dodecyl Sulphate Polyacrylamide Gel Electrophoresis (SDS-PAGE)

Kiwi extract was mixed with NuPAGE^®^ LDS sample buffer (Invitrogen, Carlsbad, CA, USA) with the addition of 0.05 M dithiothreitol (DTT) and heated at 70 °C for 10 min. Molecular weight (MW) markers (Cat. No. LC5925, Invitrogen) were used to estimate sample MW. Electrophoresis was carried out at 100V for one hour by using NuPAGE^®^ Novex Bis-Tris 12 well Gel 4-12% (Invitrogen).

Proteins were transferred to Immobilon-P transfer membrane (Millipore, Bedford, MA, USA) by electroelution at 30 V for one hour and stained with ImperialTM Protein Stain (Pierce, Rockford, IL, USA) to test the quality of transfer and the total protein content.

### 2.7. IgE Immunoblotting

Immobilon-P transfer membranes (Millipore) containing blotted protein were cut into strips prior to immunolabelling with sera from patients. Sera from two patients not allergic to kiwifruit were used as negative controls. After blocking the membranes with 3% bovine serum albumin (BSA) in Tris-buffered for 1h at room temperature, strips were incubated with diluted sera 1:5 for another hour. The strips were then washed with PBS three times for 15 s each and incubated for 1h with ^125^I-labelled goat anti-human IgE (DiaMed, Windham, ME, USA) diluted in PBS-Tween 20 plus 1% BSA and 10% normal goat serum. Blotted membranes were washed with PBS three times, exposed to Kodak Biomax Imaging Film (Carestream Health Inc., Rochester, NY, USA), and developed seven days later.

Mass spectrometry/mass spectrometry (MS/MS) sequencing analysis was performed at the Wistar Institute Proteomics Facility (Philadelphia, PA, USA) using microcapillary reverse phase high-performance liquid chromatography (HPLC) nano-spray tandem mass spectrometry on a ThermoFinnigan LTQ quadrupole ion trap mass spectrometer. The MS/MS spectra were run against a sequence database using the program SEQUEST.

### 2.8. *In Vitro* Component Resolved Diagnosis

Sera of 17 kiwifruit allergic patients were analyzed by an allergen microarray assay (ISAC, Immuno Solid-Phase Allergen Chip, Phadia Multiplexing Diagnostics, Vienna, Austria) with four native kiwifruit allergens (nAct d 1, nAct d 2, nAct d 5 and rAct d 8).

## 3. Results

### 3.1. Patients

Twenty four children (nine girls and 15 boys) with a median age of eight years (range: 3–12 years) were recruited for the study. Serum samples from seventeen children were obtained.

Regarding clinical manifestations, 13 children experienced oral symptoms, nine patients presented urticaria, two patients developed rhinoconjunctivitis, two patients presented with dyspnea and wheezing, four patients reported facial angioedema, and five patients abdominal pain and vomiting. Nine children showed systemic reactions involving more than one organ system.

Seventeen children (71%) developed clinical manifestations on their first known exposure. Fifteen children (63%) were younger than four years of age when they first reacted to kiwifruit. Thirteen suffered from asthma, 16 from allergic rhinitis and 14 from atopic dermatitis. The majority of children (75%) were sensitized to dust mites and about one-third (37%) were sensitized to grass pollen. Fifteen children suffered from other food allergies (most frequently egg and fish). Clinical data, SPT results, and specific IgE to kiwifruit determined by CAP and ISAC, are summarized in Table 1.

**Table 1:** 

No.	Sex	Age (Years)	Clinical Manifestations	Other Food Allergies	Other Allergies	Other Allergens	Prick Kiwi (mm)	Prick-Prick Kiwi (mm)	Kiwifruit Specific IgE (kU/L)									
										**Act d 1**	**Act d 2**	**Act d 5**	**Act d 8**	**Pru p 1**	**Pru p 3**	**Cor a 1.04**	**Cor a 8**	**Cor a 9**
**1**	M	11	Abdominal pain, vomiting		Rhinitis	Dust mites, timothy grass pollen	4 × 4	4 × 3	ND	0	0	0	0	2.35	0	0	0	0
**2**	M	5	Itchy throat, lip swelling	Strawberry	Asthma, rhinitis, atopic dermatitis	Dust mites, timothy grass pollen, cat dander	3 × 3	4 × 3	ND									
**3**	F	8	Swelling of lips and eyelids, vomiting, abdominal pain	Egg and fish	Asthma, atopic dermatitis	Dust mites	9 × 5	11 × 7	10.8	3.81	4.08	0	0	0	3.06	0	4	0
**4**	M	6	Generalized hives and erythema		Atopic dermatitis		8 × 2	3 × 4	ND	1.83	0	0	0	0	1.5	0	0	0
**5**	F	5	Hives on the face, lip swelling		Asthma, atopic dermatitis	Dust mites	ND	6 × 3	<0.35									
**6**	F	12	Itchy mouth		Rhinitis, conjunctivitis	Dust mites	10 × 9	5 × 4	ND	4.2	0	0	0	2.24	0	0	0	0
**7**	M	12	Hives, itchy throat		Asthma, rhinitis, atopic dermatitis	Dust mites, timothy grass pollen, herbaceous	4 × 4	11 × 7	ND	2.28	0	0	0	2.44	0	0	0	0
**8**	M	9	Lips swelling	Seafood	Rhinitis, atopic dermatitis	Dust mites	10 × 6	20 × 12	<0.35	5.54	0	0	0	2.49	0	0	0	0
**9**	F	7	Hives and erythema, wheezing, moderate dispnea	Egg	Asthma	Dust mites	7 × 7	ND	9.22									
**10**	M	11	Generalized hives and erythema		Rhinitis	Dust mites	7 × 6	7 × 6	<0.35	0	0	0	0	1.89	0	0	0	0
**11**	M	8	Vomiting, abdominal pain	Fish, egg and peanut	Asthma, rhinitis	Dust mites, cat dander	12 × 7	17 × 10	17.8	9.81	0	0	0	2.31	0	0	0	0
**12**	F	10	Facial swelling, contact urticaria	Tree nuts	Asthma, rhinitis		ND	5 × 9	0.97	0	0	0	0	2.61	0	0	0	22
**13**	M	3	Perioral erythema and edema	Cow, milk, protein	Atopic dermatitis		10 × 6	10 × 7	<0.35	0	0	0	0	1.69	0	0	0	0
**14**	F	6	Itchy throat and mouth, lips swelling	Egg, peanut, hazelnut, walnut and peanut			5 × 3	14 × 4	0.82	0	0	0	0	1.67	0	0	0	0
**15**	F	12	Hives and lips swelling		Asthma, rhinitis	Dust mites, timothy grass pollen, birch pollen, herbaceous, cat dander	13 × 8	ND	4.28	3.27	0	0	0	1.68	0	1.7	0	0
**16**	M	9	Generalized hives, vomiting and diarrhea	Egg and banana	Atopic dermatitis, latex allergy	Dust mites	7 × 7	15 × 7	17.9	7.74	2.73	40.33	29.5	0	18.66	0	8.6	0
**17**	F	6	Erythema and edema of lips and ear	Egg	Atopic dermatitis		7 × 9	ND	ND	0	0	0	0	0	0	0	0	0
**18**	M	8	Eyelids swelling, red and watery eyes, nasal congestion and itching, sneezing		Asthma, rhinitis, atopic dermatitis	Dust mites, timothy grass pollen, latex	ND	5 × 7	3.33									
**19**	M	11	Lips swelling, oral itching, repetitive coughing and wheezing, dyspnea	Banana, chickpea, prawn, pea, squid, apple, pear, peach, watermelon, plum, cherry	Asthma, rhinitis, atopic dermatitis	Dust mites, cat and dog dander	ND	7 × 7	ND									
**20**	M	7	Itchy throat and mouth, lips swelling, throat tightness	Egg	Rhinitis, conjunctivitis	Timothy grass pollen	5 × 5	5 × 7	<0.35	0	0	0	0	0	0	0	0	0
**21**	F	9	Erythema, hives, lip swelling, oral itching		Asthma, rhinitis, atopic dermatitis	Dust mites, fungus, cat dander	12 × 7	7 × 10	ND	0	0	0	7.02	0	0	0	0	0
**22**	M	8	Lips swelling, itchy throat, red or watery eyes	Seafood	Rhinitis, conjunctivitis	Dust mites, timothy grass pollen	3 × 3	3 × 3	<0.35	0	0	0	0	1.84	0	1.4	0	0
**23**	M	3	Erythema and edema of lips and ear, oral itching	Milk, egg.	Asthma, rhinitis, atopic dermatitis	Dust mites, timothy grass pollen	ND	19 × 15	ND									
**24**	M	12	Abdominal pain, vomiting, diarrhea,	Egg, fish	Asthma, rhinitis, atopic dermatitis	Dust mites, timothy grass pollen	5 × 6	11 × 7	9.47									

### 3.2. Skin Testing and Serum Specific IgE

Skin tests were performed with commercial extracts in 19 patients, with positive results in all of them. SPT with fresh kiwifruit pulp were positive in all 21 patients tested.

Six out of 15 children (40%) tested had undetectable levels of serum kiwifruit-specific IgE (<0.35 kUA/L).

### 3.3. Detection of IgE-Binding Proteins in the Kiwifruit Extract

**Figure 1:**
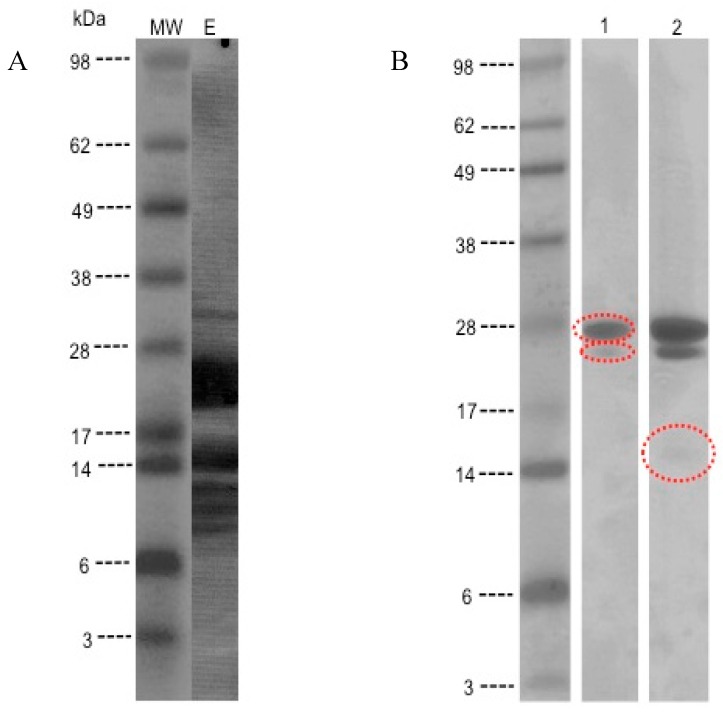
SDS-PAGE analysis for kiwi protein extract. (A) MW: Molecular weight standard and E: Kiwi extract. (B) Two lanes (1 and 2) of the gel which was sent to the sequencing facility for MS/MS analysis and identification of the proteins

Protein staining of the kiwifruit extract separated by SDS-PAGE showed several bands distributed in the range of 12 to 38 kDa (Figure 1).

**Figure 2:**
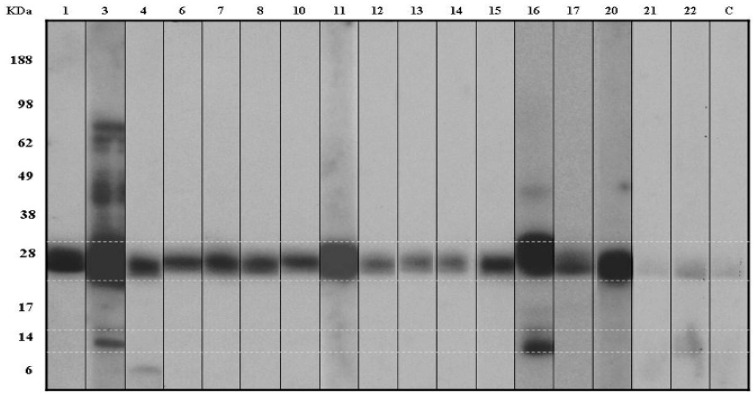
Immunolabelling with sera from 17 patients with kiwifruit allergy. Lower and upper marks indicate Act d 5 at 15 kDa and Act d 1 and Act d 2 at 28 and 24 kDa. C: negative control.

IgE-binding bands were identified at 80, 62, 40, 28, 24, 15, and 6 kDa by immunoblotting using patients’ sera. Fifteen out of 17 children tested (88%) had IgE antibodies that recognized the 28 kDa (Act d 1) and 24 kDa (Act d 2) proteins; two children (12%) had IgE that recognized a protein of 15 kDa (kiwellin) (Figure 2).

### 3.4. MS/MS Peptide Sequence Analysis

The 28 kDa, 24 kDa, and 15 kDa bands of SDS-PAGE were excised from the gel for sequence analysis. Peptides were compared with known proteins in the database. The 28 kDa protein was confirmed to be the known major allergen, actinidin (Act d 1). The 24 kDa protein corresponded to a thaumatin-like protein (Act d 2) and the third protein with MW of 15 kDa, showed 67% homology to kiwellin (Act d 5). The sequences of three proteins are indicated below (including Table 2, Table 3 and Table 4):


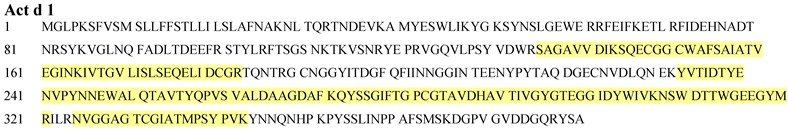


**Table 2:** Summary of peptide positions from Act d 1.

Peptide	Position
SAGAVVDIK	135-143
SQECGGCWAFSAIATVEGINK	144-165
IVTGVLISLSEQELIDCGR	166-184
YVTIDTYENVPYNNEWALQTAVTYQPVSVALDAAGDAF	233-271
QYSSGIFTG PCGTAVDHAV TIVGYGTEGG IDYWIVK	272-307
NSW DTTWGEEGYMR	308-321
NVGGAGTCGIATMPSYPVK	325-343


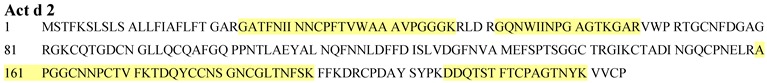


**Table 3:** Summary of peptide positions from Act d 2.

Peptide	Position
GATFNIINNCPFTVWAA AVPGGGK	24-47
GQNWIINPGAGTK	52-64
GQNWIINPGAGTKGAR	52-67
APGGCNNPCTVFK	160-172
TDQYCCNSGNCGLTNFSK	173-190
DDQTSTFTCPAGTNYK	205-220


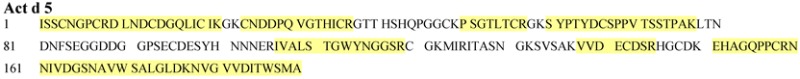


**Table 4:** Summary of peptide positions from Act d 5.

Peptide	Position
ISSCNGPCR	1-9
D LNDCDGQLICIK	10-22
CNDDPQVGTHICR	25-37
PSGTLTCR	50-57
S YPTYDCSPPVTSSTPAK	60-77
IVALSTGWYNGGSR	106-119
VVDECDSR	138-145
EHAGQPPCRN	151-159
NIVDGSNAVWSALGLDK	160-177
NVGVVDITWSMA	178-189

### 3.5. Evaluation of the ISAC

ISAC was performed in 17 patients. Results from ISAC are summarized in Table 1.

Specific IgE was detected against at least one of the four kiwifruit allergens tested in nine children (53%). nAct d 1 was the allergen with the highest frequency of recognition (eight children, 47%), followed by nAct d 2 (two children, 12%) and nAct d 8 (two children, 12%).

Two children (12%) were sensitized to latex profilin rHeb v 8; eleven children (65%) recognized proteins from peach and two (12%) from hazelnut belonging to the PR10 family (rPru p 1, rCor a 1.0401, respectively). Three children (18%) recognized peach LTP (rPru p 3) and two children (12%) recognized hazelnut LTP (rCor a 8).

## 4. Discussion

There has been an increase in the incidence of kiwifruit allergy in the last few years with new cases more often affecting younger infants [4]. Kiwifruit is becoming one of the more common causes of food allergy in Mediterranean countries such as France, where it is the third most common food allergen in children after milk and egg, affecting 9% of children [17]. In Portugal, Vieira *et al.*, found that kiwifruit is the most allergenic fruit, eliciting allergic symptoms in 60% of a pediatric cohort, followed by peach (50%) [18]. In our clinical practice, green kiwifruit seems to be the main fruit causing allergic reactions in children, far more often than Rosacea fruits.

More than half of children were younger than four years of age when they first showed clinical manifestations following kiwifruit ingestion and most of them reacted on their first known exposure, suggesting either unknown prior sensitization to kiwifruit or to another cross-reactive protein source. Regarding the latter, two children from our series were sensitized to latex profilin rHeb v 8, although only one of them presented symptoms after manipulating latex products; eleven children had IgE that bound to proteins from peach and two from hazelnut belonging to the PR10 family (rPru p 1, rCor a 1.0401, respectively). Three children recognized peach LTP (rPru p 3) and two children recognized hazelnut LTP (rCor a 8). Although these data might suggest a certain degree of cross-reactivity, a low degree of sequence homology between these allergens has been described [13] and, indeed, we could not demonstrate inhibition to peach allergens by ISAC inhibition with kiwifruit extract (data not shown). None of the children presented symptoms after ingesting peach or hazelnut, although Vieira *et al.* [18] have reported clinical manifestations with peach in a minority of kiwifruit allergic children, who recognized rPru p 3 as well.

Regarding clinical manifestations, symptoms were confined to the oral mucosa in half of our subjects, a lower percentage compared to previous studies in adult populations [8,9]. In fact, Aleman *et al.* [8], found that 100% of Spanish adults suffering from kiwi allergy presented with oral allergy syndrome, but only three out of the 42 patients studied developed anaphylaxis. We found that 13% of our pediatric patients experienced anaphylaxis suggesting that Spanish children are more likely to develop severe reactions after kiwifruit ingestion than adults, as previously described [4,8,9]. This could be related to the fact that in this population, sensitization to kiwifruit is not related to cross-reacting allergens from pollen, which is frequently described in older patients [8]. On the other hand, the potential gastrointestinal route of sensitization could expose different allergens or combinations of allergens from kiwifruit that may elicit more severe clinical manifestations in children.

Two proteins of 28 kDa and 24 kDa corresponding to Act d 1 and Act d 2, respectively, were identified as major allergens in our pediatric population. Actinidin (Act d 1) was described for the first time by Pastorello *et al.* [5,6] in an adult population presenting with oral allergy syndrome, and is considered one of the main allergens from kiwifruit, [9] although it does not seem to be as relevant in all populations, as reported by Lucas *et al.* in the United Kingdom [19]. Initially correlated with mild symptoms [6], more recently Act d 1 has been detected in patients with more severe reactions [9,20]. In agreement with this observation, five of the eight children from our study that recognized Act d 1 presented systemic manifestations after kiwifruit ingestion.

A third protein of 15kDa was recognized by two children who developed systemic reactions after kiwifruit ingestion. It was identified as kiwellin (Act d 5). Kiwellin may undergo proteolytic processing by actinidin, leading to two fragments named KiTH (16 kDa) and kissper (4 kDa). Based on its molecular weight, it seems that both children in our study recognized the N-terminal sequence, KiTH, which has been demonstrated to show IgE binding ability [21]. More studies on pediatric populations are needed to establish the potential association among Act d 5 and more severe clinical manifestations.

On the other hand, in our sample, five children were not sensitized to airborne allergens, 10 children were sensitized to pollen (mainly grass pollen, and only one patient to birch pollen) and the remaining nine children were sensitized to indoor allergens such as dust mites and/or cat dander. By SDS-PAGE immunodetection (Figure 2), we did not find significant differences in allergen recognition between children sensitized to pollen and indoor allergens or to no airborne allergens at all.

It would be interesting for further studies to compare clinical manifestations and patterns of recognition among atopic and non-atopic children, as previously described for adult population.

Regarding the diagnosis of kiwifruit allergy, SPT with commercial extracts and *in vitro*-specific IgE determination have shown low sensitivity and specificity [8,10,12]. A prick-prick test with fresh kiwifruit is more sensitive, but has low specificity [4,8,10,12]. Lack of relevant kiwifruit allergens in some commercial extracts may explain these differences. Also, the protease activity of Actinidin, may cause protein degradation during the process of extraction [22].

Interestingly, all children included in our study showed positive SPT with the commercial extracts used, thus making SPTs as sensitive as prick-prick skin testing with fresh kiwifruit for the diagnosis, contrary to what Aleman *et al.* [9] reported previously with the same extracts in an adult population. This could be due to improvement in the quality of the kiwifruit extract over the last few years, or differences in the IgE antibody response between children and adults. On the other hand, six patients did not show serum kiwifruit-specific IgE either by immunoCAP or immunodetection. This could be related to a potential sensitization to seed allergens such as Act d 12, not included in kiwifruit extracts, although for SDS-PAGE immunoblot detection this explanation seems less plausible, since kiwifruit extracts included pulp and seeds. It has been described that conditions such as the ripening stage and the extraction method influence the composition and protein concentration of green kiwifruit extracts [23].

In order to rule out the possibility of less IgE reactivity to kiwifruit secondary to heat treatment and SDS protein denaturation, a dot-blot study was performed and it was also found to be negative (data not shown).

The sensitivity of *in vitro* diagnostics has improved with the application of a panel of individual allergens from kiwifruit [10,19]. Bublin *et al.* [10] suggested that the application of component-resolved diagnostic reagents enables the classification of patients in different reactors groups. However, in our study, only nine of seventeen children (53%) showed positive results in ISAC. Eight of them recognized nAct d 1. Two children also had IgE antibodies that bound to nAct d 2, while one patient did not show positivity to nAct d 1 or nAct d 2, but had IgE to rAct d 8 (patient 21). Some factors that might influence the lower sensitivity of ISAC compared with *in vivo* tests, may be the smaller number of allergens tested in ISAC and modification of the exposed antigenic epitopes due to the assay conditions, as previously described for Act d 5 [24].

In summary, Spanish allergic children develop systemic reactions following kiwifruit ingestion more frequently than adults. Act d 1 and Act d 2 are major allergens in the Spanish pediatric age group. SPT and prick-prick tests showed greater sensitivity for the diagnosis of kiwifruit allergy than serum-specific IgE determination.
